# Involvement in Multiple Race Events Among International Para and Non-disabled Swimmers

**DOI:** 10.3389/fspor.2020.608777

**Published:** 2021-01-28

**Authors:** Julien Schipman, Guillaume Saulière, Bryan Le Toquin, Andy Marc, Nicolas Forstmann, Jean-François Toussaint, Adrien Sedeaud

**Affiliations:** ^1^Institut de Recherche Bio-Médicale et d'Épidémiologie du Sport (IRMES), EA 7329, Institut National du Sport, de l'Expertise et de la Performance (INSEP), Paris, France; ^2^Institut de Recherche Bio-Médicale et d'Épidémiologie du Sport (IRMES), Université de Paris, Paris, France; ^3^Centre d'Investigations en Médecine du Sport (CIMS), Hôtel-Dieu, Assistance Publique - Hôpitaux de Paris, Paris, France

**Keywords:** Paralympic swimmers, impairment classification, disabled athletes, network analysis, specialization, age

## Abstract

International elite Para swimmers form a large portion of the overall multi-medalist winning population. For the highest performing Para swimmers, world class performances were achieved across different strokes. The aim of this study was to quantify the level of involvement across different events and to examine this in relation to the level of performance. The performances in swimming speed of the top 100 females and males for both Para- and non-disabled swimmers were collected in 11 race events between 2009 and 2019 (4,400 performances for 307 Para females and 365 Para males, 605 non-disabled females, and 715 non-disabled males). We tallied the number of events in which each swimmer was involved. Swimmers were grouped according to the total number of race events in which they participated. Then the association between involvement and level of performance was investigated. Para swimmers with impairment from classes seven to 14 were involved in a range of race events across different strokes. The most common combination for both Para and non-disabled athletes was over similarly distanced races of the same stroke (50 and 100 m freestyle). The more race events in which Para swimmers involved, the higher the level of performance that was achieved. This trend can partially be explained by the less concentrated competition pool for Para swimmers compared to able-bodied swimmers. Para swimmers with minimal and no physical impairment perform in multiple race events more often than able-bodied swimmers. Fewer Para swimmers at the international level and a less concentrated competition pool could explain these differences.

## Introduction

Para swimming was first introduced into the Paralympics Games in Rome in 1960. It has one of the highest medal counts at the Paralympics Games alongside track and field and cycling (Tweedy and Howe, 2010). As a result of public support, geopolitical issues, and the increasing media coverage of the Paralympic Games, the Para athletes' performances have strongly evolved in recent years and are consistently improving (Fulton et al., 2009; Lepers et al., 2012; Grobler et al., 2015; Burkett et al., 2018) in comparison to non-disabled athletes who are reaching their performance limits in many sports (Berthelot et al., 2015; Marck et al., 2017, 2019). Consequently, Paralympic swimming performances have progressed more than Olympic swimming performances over the past two decades for the majority of impairment classifications (Burkett et al., 2018).

Another feature of Para swimming race events is that they seem to provide an opportunity for Para swimmers (PS) to win across several distances and strokes despite a fewer race events available compared to non-disabled swimmer (NDS). The Para swimming race events includes four different strokes (freestyle, butterfly, backstroke, and breaststroke) swum over only four distances (50, 100, 200, and 400 m) with 14 impairment classifications. Trischa Zorn is the world's most successful female Paralympic swimmer, winning 37 gold medals for a total of 55 medals between 1980 and 2004 over all types of stroke styles from 50 to 400 m. She is followed by Beatrice Hess who won 20 gold medals between 1984 and 2004 in freestyle, butterfly, backstroke, the individual medley (IM) over several distances from 50 to 200 m. In contrast, among NDS, the best multi-medalist (Michael Phelps) won 23 gold medals between 2004 and 2016 in freestyle, butterfly, the IM, and relays on several distances from 100 to 400 m. From the junior national level (Stewart and Hopkins, 2000) to sub-elite level (Dormehl and Williams, 2016), high level non-disabled swimmers tend to perform in one or two race events with the most common overlap in races consisting of the 50 and 100 m freestyle.

It is well-known that performance level is strongly related to age, which varies according to sport, event, and sex among non-disabled (Berthelot et al., 2012) and Para athletes. Differences also exist between impairment type (Schipman et al., 2019). In swimming, non-disabled male athletes reach their performance peak between 20 and 24 years old, varying based on distance. Female non-disabled athletes usually reach this peak 2 years younger (Allen and Hopkins, 2015; Knechtle et al., 2016; Berthelot et al., 2019). The difference between sexes could be explained by the earlier onset of puberty in females compared to males (Baxter-Jones and Maffulli, 2002). Among international PS, age has not been studied and should be considered in order to be quantified and optimized.

The main differences between PS and NDS are the impairment classification based on disability (Burkett et al., 2018) and the maximum swimming distance of only 400 m for PS. In Para swimming, there is a wide classification range of physical (classes from 1 to 10), visual (11 to 13) and intellectual impairments (14) (World Para Swimming, 2017. World Para Swimming classification rules and regulations. Bonn: International Paralympic Committee). Among the physical and visual classifications, greater values mean less impairment. The PS with minimal or motor impairments (10), slight visual impairments (12–13) and intellectual disabilities (14) tend to be the fastest, partially due to better propulsion and also to lower active drag in the water than others classes (Burkett et al., 2018; Payton et al., 2020).

The long careers of Para swimmer (PS) multi-medalists as Trisha Zorn or Beatrice Hess over several stroke styles and distances and the various classes of impairment have led to questions regarding multi-race event involvement at a high-performance level. No previous study has been addressed on this aspect of Para swimming competition.

The aim of this study was to investigate the specificities of the international Para- and non-disabled swimmers according to their involvement in multiple race events, performance level, impairment class among Para swimmers, and age. Para swimmers may be involved in a more diverse set of race events than NDS. Indeed, the fewer concentration of PS in each race event and the large number of impairment classifications might increase the opportunity to win more in the few events that are available. In comparison, the NDS who would be more numerous with more races available leading to a specialization on a race event in order to be able to reach an international podium.

## Methods

### Data Collection

The performances of the top 100 Para- (PS) and non-disabled (NDS) swimmers were collected from 2009 to 2019 from all International Paralympic Committee (IPC) competitions and International Swimming Federation (FINA) competitions, totaling 44 race events. Data were collected from publicly available, official swimming websites: www.ipc.org for PS and www.fina.org for NDS. Before 2009, data were unavailable on the IPC website. The long course race events (50 m swimming pool) collected were the 50, 100, 200, and 400 m freestyle; the 50 and 100 m butterfly; the 50 and 100 m backstroke; the 50 and 100 m breaststroke; and the 200 m IM. The short course race events (25 m swimming pool) were excluded. A total of 4,400 swimming performances were extracted for 307 Para females and 365 Para males, 605 non-disabled females and 715 non-disabled males. Names, stroke, age, sex, and impairment classification were also collected.

Performance levels were derived from the race times by converting them into average speeds (m.s^−1^). Speeds were normalized and calculated as a percentage (%) of the fastest speed by type of event and sex for both PS and NDS.

### Concentration of Competitors

In order to quantify the mean performance level and the gap to the best performance of each PS and NDS, we pooled all race events of the same stroke and calculated the difference between the mean performance compared with the best performance for each swimmer by stroke. Additionally, we created a category that included all strokes and calculated the mean performance level. The mean level of performance was plotted by sex and stroke for PS and NDS (Figure 3).

### Involvement in Multiple Race Events

To quantify the involvement of athletes in multiple race events, we recorded the number of events in which the swimmer took part across the study period. A bipartite network with two sets of nodes: (i) swimming race events (11 nodes) and (ii) swimmers (1,992 nodes) was created. Only links between different sets were allowed. Four representations of the network are illustrated in Figure 2, drawn using the Kamada Kamai algorithm (Kamada and Kawai, 1989), stratified by sex for both PS and NDS. From these networks, Table 1 reports the adjacency matrix for the percentage of swimmers who participated in each combination of two race events. An analysis was performed depending on the stroke, regardless of the distance.

**Table 1:** Percentage (%) of female (A) and male (B) swimmers who perform each combination of two race events related to the total number of swimmers in these two events.

			**Freestyle**				**Butterfly**		**Backstroke**		**Breaststroke**		**Individual Medley**	
			**50**	**100**	**200**	**400**	**50**	**100**	**50**	**100**	**50**	**100**	**200**	
		**%**	**Non-disabled**											
**(A) FEMALE**														
Freestyle	50	Para	/	39.9	10.5	2.6	22.7	13.6	8.1	6.4	1	0.5	3.6	Non-disabled
	100		57.5	/	28.2	5.3	21.2	16.3	8.1	9.9	1	1.6	8.1	
	200		22	43.9	/	25.8	6.4	8.1	3.1	5.3	0	1	9.9	
	400		31.6	43.9	39.9	/	2	2	1	2	0	0.5	4.2	
Butterfly	50		37	28.2	19.1	24.2	/	41.8	7.5	3.6	0.5	0	3.1	
	100		35.1	44.9	32.5	36.1	39.9	/	4.2	3.6	0	0.5	6.4	
Backstroke	50		29	22	13.6	21.2	39.9	22	/	44.9	0.5	0	4.2	
	100		34.2	43.9	30.7	33.3	21.2	30.7	29.9	/	0.5	0	5.8	
Breaststroke	50		22	16.3	8.7	13	28.2	17	23.5	8.7	/	41.6	2.6	
	100		25	25.8	17.7	16.3	16.3	26.6	14.3	17	35.1	/	6	
Individual Medley	200		33.3	48.2	37.9	39.9	28.2	50.4	22.7	37	24.2	42.9	/	
			Para											
**(B) MALE**														
Freestyle	50	Para	/	32	2.6	0	16.3	2.6	3.6	0.5	1	0.5	0.5	Non-disabled
	100		55	/	17.9	2.6	13.8	8.2	3.7	3.1	0.5	0	2.1	
	200		14.9	34.2	/	23.5	0	0.5	0.5	4.2	0	0	8.1	
	400		29	42.9	32.5	/	0	0.5	0.5	1	0	0	4.2	
Butterfly	50		36.1	27.4	11.1	18.3	/	27.4	4.7	5.3	1.5	1	2.6	
	100		35.1	50.4	24.2	30.7	29.9	/	3.6	4.7	0.5	1	6.4	
Backstroke	50		27.4	18.3	8.1	14.9	29	16.3	/	34.2	1.5	0.5	3.1	
	100		30.7	40.9	22	25	18.3	37	29	/	1.5	0.5	7	
Breaststroke	50		15.6	10.5	4.2	5.3	19.1	7	13	5.8	/	36.1	0.5	
	100		14.9	14.3	12.4	9.3	10.5	15.6	7.5	11.1	30.7	/	3.6	
Individual Medley	200		30.7	44.9	36.1	34.2	15.6	37.9	17.7	40.9	12.4	26.6	/	
			Para											

*The left diagonal corresponds to the Para swimmers and the right diagonal to the non-disabled swimmers. The color gradient depicts the strength of the overlap between the two events: Navy blue (very low), Red (high)*.

In order to investigate a potential association between the performances of swimmers and their involvement in multiple race events, we grouped swimmers based on the number of nodes from set (i), where they had a link. Balanced groups are illustrated in Table 2 and were obtained by splitting the distribution of the number of race events in which swimmers competed. For Para swimmer, we split the distribution into five groups: swimmers involved in only one race event [1,2], swimmers involved in two race events [2,3], swimmers involved in three to four race events [3,5], and swimmers involved in five to 11 race events [5,11]. As the distribution for swimmers involved in multiple race events was different among NDS, only three groups were established: [1,2], [2,3], and [3,8]. In order to investigate the relation with the performance levels and the gap to best performance, we calculated the mean performance levels of each swimmer by stroke according to their involvement group. Once again, we created a category that included all strokes together.

**Table 2:** Number of performances by stroke style (all distances combined) and involvements in group events for Para and non-disabled female's and male's swimmers.

	**Para**				**Non-disabled**		
	**[1,2)**	**[2,3)**	**[3,5)**	**[5,11]**	**[1,2)**	**[2,3)**	**[3,8]**
**Female**							
All	93	86	171	382	331	242	202
Freestyle	18	25	53	91	107	74	76
Butterfly	10	19	29	85	45	41	55
Backstroke	26	18	37	73	54	52	32
Breaststroke	39	23	27	59	71	57	11
Individual Medley	0	1	25	74	54	18	28
**Male**							
All	140	93	192	343	427	266	143
Freestyle	36	26	55	89	141	90	52
Butterfly	18	16	40	80	61	56	40
Backstroke	36	14	36	69	75	48	26
Breaststroke	50	31	33	39	86	56	5
Individual Medley	0	6	28	66	64	16	20

### Statistical Analysis

Data were reported as mean ± standard deviation. A one-way ANOVA with a Tukey *post-hoc* test was performed to test for performance differences between groups according their involvement in multiple race events (based on the splitting method described above). Age differences between PS and NDS according to sex, stroke, and distance were also tested with a one-way ANOVA and Tukey *post-hoc* test. In addition, the distribution of impairment types according to sex, stroke, and distance was analyzed. All statistical analyses were performed using R (version 3.6.2; The R Foundation for Statistical Computing, Vienna, Austria). Results are considered significant at an alpha of 0.05.

## Results

### Descriptive Results

**Figure 1:**
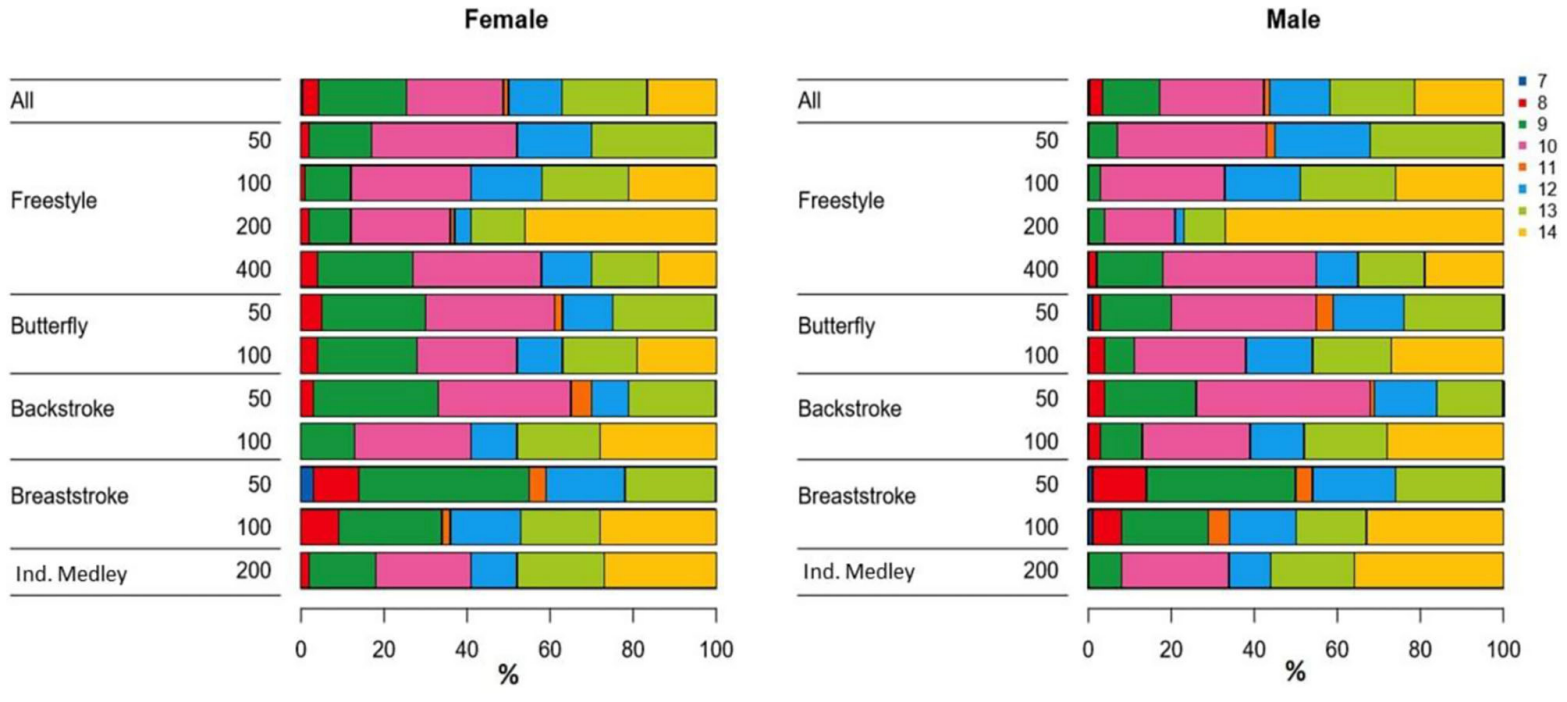
Distribution (%) of female and male Para swimmers by impairment classification and stroke. The colors correspond to the impairment classification from the class 7 (Navy Blue) to class 14 (Yellow).

Swimmers' impairment classifications, according to stroke and sex, are illustrated in Figure 1. For both sexes, they ranged from classifications 7–14. Para swimmers from classifications 1–6 were not represented. When all strokes and distances were combined, the most common impairment classification among the top 100 female swimmers was Class 10 = 23.4%. This was followed by Class 9 = 21.2%, Class 13 = 20.5%, and Class 14 = 16.6%. The top classes among males were Class 10 = 25.1%, Class 14 = 21.5, Class 13 = 20.3%, Class 12 = 14.5% (Figure 1).

Female PS were significantly younger than their NDS counterparts in nine race events (Table 3). Male PS were significantly younger than their NDS counterparts in only the 200 m freestyle (21.0 ± 3.5 vs. 22.7 ± 3.0), the 50 m breaststroke (22.9 ± 5.4 vs. 24.2 ± 3.1), and the 200 m IM (21.7 ± 3.5 vs. 22.6 ± 2.5) (Table 3).

**Table 3:** Mean age (± *SD*) of the Top 100 swimmers by event for Para and Non-disabled females and males.

		**Female**			**Male**		
		**Para**	**Non-disabled**	***p***	**Para**	**Non-disabled**	***p***
Freestyle	50	21.3 ± 3.9	23.8 ± 4.4	^*^	23.3 ± 3.7	23.9 ± 3.3	
	100	20.7 ± 3.5	22.5 ± 3.7	^*^	22.7 ± 3.9	23.4 ± 2.9	
	200	20 ± 3.3	21.2 ± 3.1	^*^	21 ± 3.5	22.7 ± 3	^*^
	400	20.6 ± 4.7	20.8 ± 3.5		21.5 ± 3.8	22.1 ± 3	
Butterfly	50	20.6 ± 3.8	22.9 ± 4	^*^	23.2 ± 4.3	23.6 ± 3.7	
	100	20.6 ± 3.8	22.3 ± 3.8	^*^	22.5 ± 3.7	23.4 ± 3.3	
Backstroke	50	19.7 ± 4.1	22.1 ± 4.3	^*^	22.9 ± 5.4	23.3 ± 3	
	100	20.2 ± 3.6	21.7 ± 3.9	^*^	22.8 ± 4.5	22.9 ± 3.4	
Breaststroke	50	20.3 ± 4.3	21.7 ± 3.9	^*^	22.9 ± 5.2	24.2 ± 3.1	^*^
	100	20.3 ± 3.9	22 ± 3.7	^*^	23.4 ± 4.6	23.8 ± 3.2	
Individual medley	200	20.2 ± 3.5	20.8 ± 3.1		21.7 ± 3.5	22.6 ± 2.5	^*^

* *Indicates a significant age difference (p ≤ 0.05) between Para and Non-disabled swimmers for female and male*.

### Involvement in Multiple Race Events

**Figure 2:**
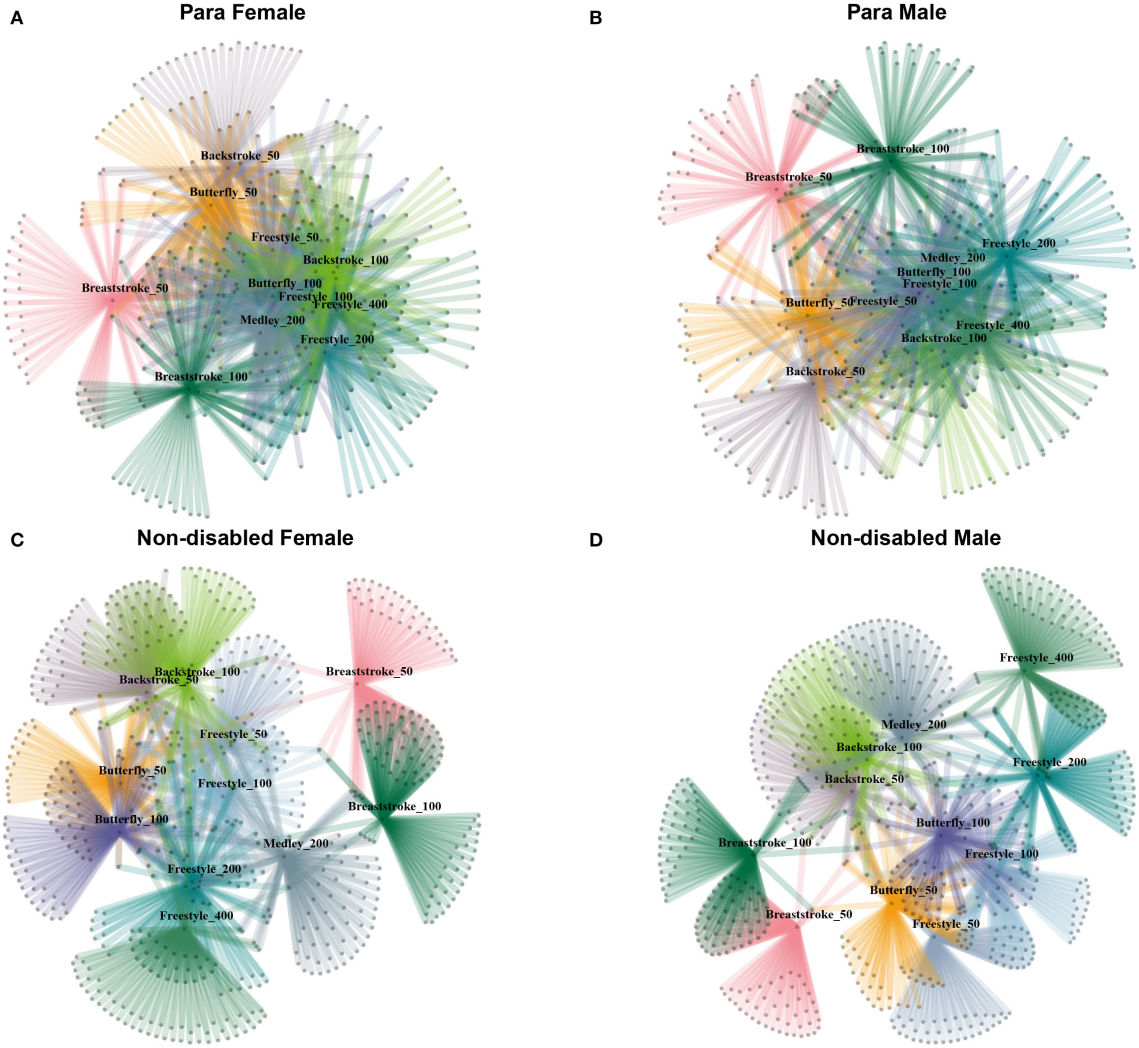
Bipartite network representation of the involvement in multiple events for Para female **(A)**, Para male **(B)**, non-disabled female **(C)**, and non-disabled male **(D)**. Labeled nodes represent types of event (stroke and distance); non-labeled ones represent swimmer.

Female PS swam on average 3.5 ± 2.6 race events and NDS swam 1.7 ± 1.1 race events. Male PS swam 3.0 ± 2.2 race events while NDS swam 1.5 ± 0.8 race events (Figure 2).

The race events with the greatest overlap of swimmers were the 50 and the 100 m freestyle, where 57.5% of female PS and 55.0% of the male PS participated in both compared with 39.9% for female NDS and 32.0% for male NDS (Table 1). For both male and female PS, breaststroke had the least overlap with other strokes. The majority of NDS involved in breaststroke swam it exclusively (Figure 2 and Table 1).

Regarding the PS in the 200 m IM, 50.4% of women also swam the 100 m butterfly and 48.2% the 100 m freestyle (Table 1); 44.9% of men also swam the 100 m freestyle and 40.9% the 100 m backstroke (Table 1). For NDS in the IM, 8.1% of women also swam the 100 m freestyle and 9.9% the 200 m freestyle; 2.1% of men also swam the 100 m freestyle and 8.1% the 200 m freestyle (Table 1).

### Performance Level

**Figure 3:**
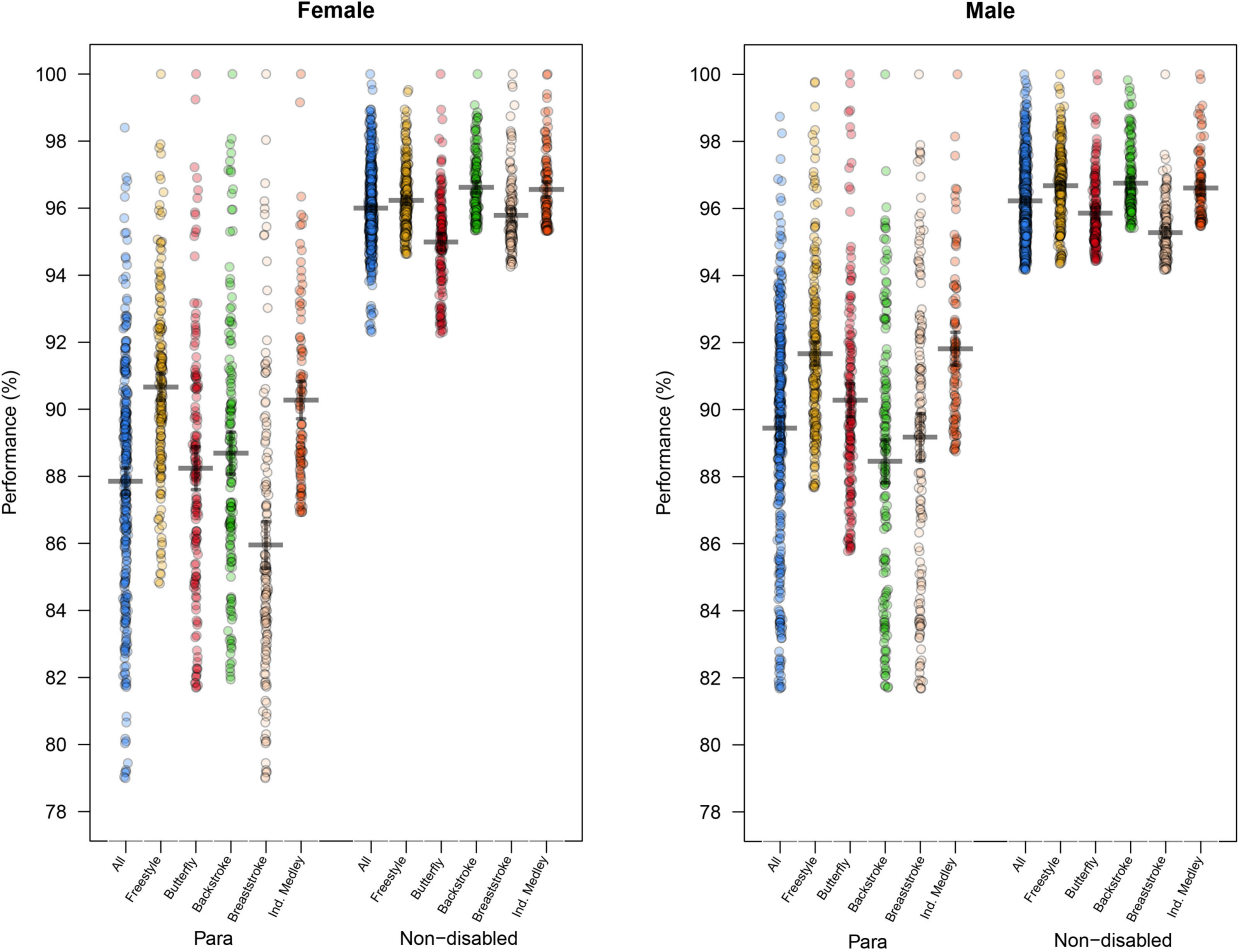
Competition Performance Level: mean (± *SD*) performance levels of each swimmer in % compared to the best performance by events according to sex and stroke for Para swimmers and non-disabled swimmers.

For female PS, the mean performance level for all strokes was 87.8 ± 3.6% when compared with the best performances. The stroke where the mean level had the smallest difference compared with the best performance was freestyle with a performance level of 90.7 ± 2.7%. For female NDS, the performance level for all strokes was at 96 ± 1.1% when compared with the best performances. The smallest difference between the mean and best performances was for backstroke with a performance level of 96.6 ± 1% (Figure 3).

For male PS, the performance level for all strokes was 89.5 ± 3.3% when compared with the best performances. The smallest difference between the mean and best performances was for the IM with a performance level of 91.8 ± 2.4%. For male NDS, the performance level for all strokes was 96.2 ± 1.1% when compared with the best performances. The smallest difference between the mean and best performances was for backstroke with a performance level of 96.7 ± 1.0% (Figure 3).

**Figure 4:**
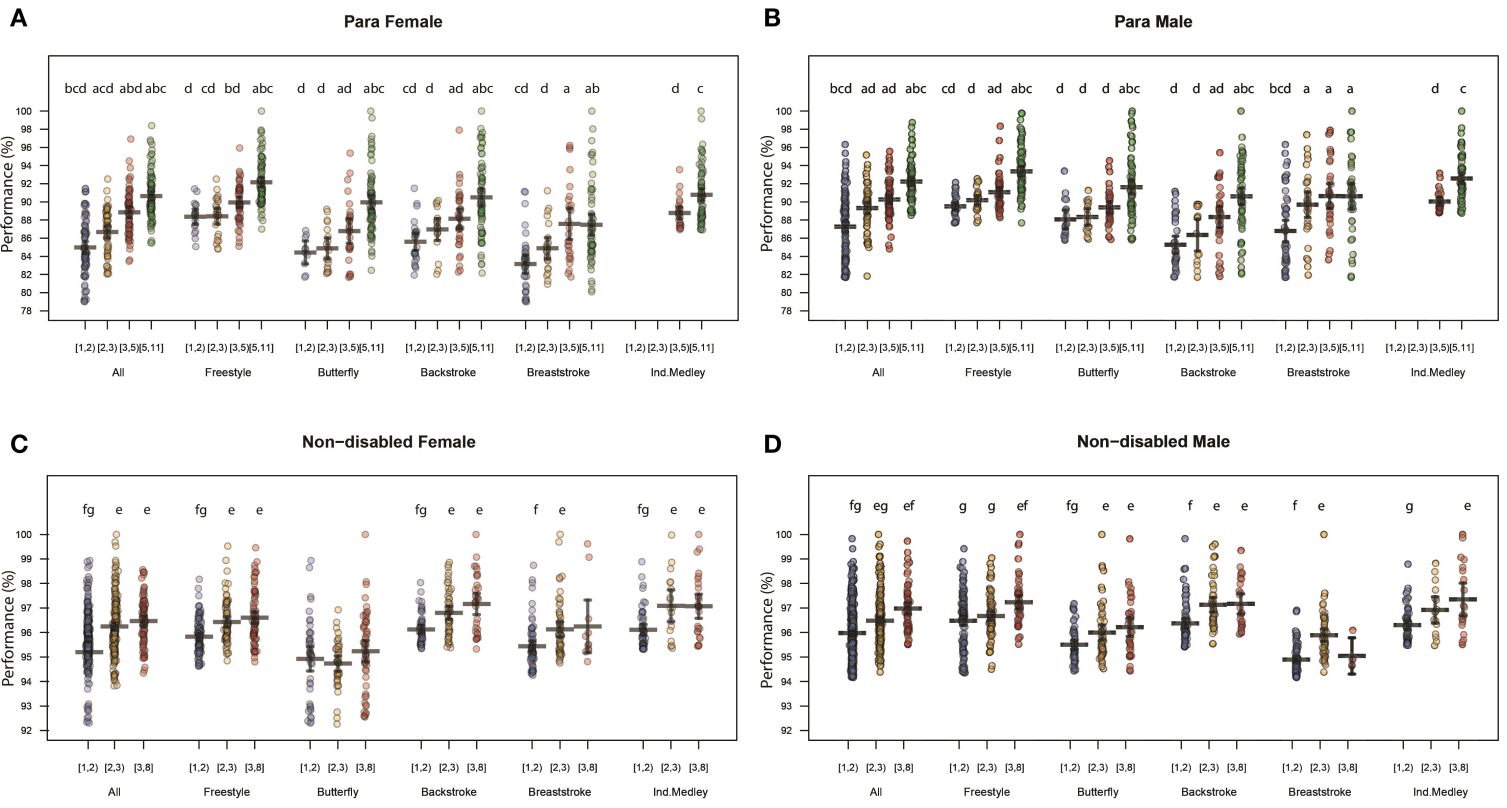
Mean performance (± *SD*) as a percentage (%) of the best performance based on the number of events in which swimmers involved, by stroke for Para female **(A)** and Para male **(B)**, non-disabled female **(C)**, and non-disabled male **(D)** swimmers. A letter indicates a significant performance difference between 2 groups of events (*p* < 0.05). The following letters correspond to the Para swimmers' group based on number of events: ^a^ [1,2); ^b^ [2,3); ^c^ [3,5); ^d^ [5,11]. The following letters correspond to the non-disablec swimmers' group based on number of events: ^e^ [1,2); ^f^ [2,3); ^g^ [3,8].

When all events were combined, we found that participating in more race events was significantly associated with higher performance levels for female PS (Figure 4A). There was a significant increase in performance levels between the group that swam one race event ([1,2): 85 ± 3.2%), two race events ([2,3): 86.7 ± 2.5%), three or four race events ([3,5): 88.8 ± 2.5%), and more than five race events ([5,11]: 90.6 ± 2.5%). For the male PS (Figure 4B), the performance levels for the different groups of race event numbers were: 87.7 ± 3.4% for [1,2), 89.3 ± 3.4% for [2,3), 90.3 ± 2.0% for [3,5), and 92.2 ± 2.1% for [5,11] with no significant difference between group [2,3) and group [3,5].

When all race events were combined for female and male NDS (Figures 4C,D), they had significantly higher performance levels when they were involved in two or more race events. The performance levels for female NDS in the different event groups were 95.6 ± 1.0% for [1,2), 96.1 ± 1.2% for [2,3), and 96.5 ± 1.0% for [3,8] (Figure 4C) with a significant increase present between [1,2) and [2,3). For male NDS, the performances significantly increased between the event groups, with performance levels of 96.0 ± 1.1% for [1,2), 96.5 ± 1% for [2,3), and 97.0 ± 0.9% for [3,8] (Figure 4D).

## Discussion

This study was the first to quantify the involvement across multiple race events for the female and male international PS and NDS. The main findings of the present study show that:

– As a consequence of the sampling method, this study focused on the less impaired PS. The majority of the Top 100 PS were with intellectual, no or minimal motor, or slight visual disabilities with 65% of the females were from the classifications 9,10, and 13 and 70% of the males were from the classifications 10,13, and 14;
– Para swimmers with minimal or no motor impairment were involved in a wider range of race events (strokes and distances) compared to NDS, with the most common combinations found across the same stroke and similar distances;
– There is a less concentrated competition field for PS; as shown in Figure 3, the mean performance gap to the faster PS is greater on all race events compared to the NDS which are highly concentrated near to the best performance;
– Higher performance levels were present for PS with minimal or no motor impairment who took part in more race events, especially among females;
– In the majority of the race events analyzed, female PS were younger than female NDS.

### Multiple Race Event Involvement and Competition Concentration

The first finding of this study was that PS were involved in more race events than NDS. Additionally, compared to NDS, there were fewer (1/2 the competitors) PS entered in race events. Overall, females were entered in more race events than males. The majority of PS participated in more than 5 race events while NDS participated in only one or two race events, which is in accordance with the previous research on NDS (Dormehl and Williams, 2016).

Para swimmers competed in events across different strokes, which require different technical and biomechanical capabilities (Pyne and Sharp, 2014; Mooney et al., 2015). In general, swimming is characterized by a sequence of coordinated actions of the trunk and limbs. Arm movement during each of the four competitive swimming strokes comprises specific phases including various sweeps, specific to each stroke (Mooney et al., 2015). Breaststroke and butterfly have more complex arm movements and kicks than backstroke or freestyle (Bartolomeu et al., 2018), but cannot be reduced to these characteristics alone as coordination, breathing, and other movements need to be considered as well (Seifert et al., 2014).

The network analysis visualization allows a good understanding of the combinations between PS and NDS. Based on the results, NDS appeared to be stroke specialists (Stewart and Hopkins, 2000; Dormehl and Williams, 2016) while this observation seemed less prevalent among PS. However, the race events with the most overlap between swimmers were race events of the same stroke (e.g., 50 and 100 m freestyle), which were also the most common combinations among able-bodied swimmers (Dormehl and Williams, 2016). Breaststroke was least frequently swum in combination with other strokes. This suggests that breaststroke swimmers are very specialized, especially for NDS, which could be due to a specialization acquired at an early age (Dormehl and Williams, 2016). The most frequent combinations for PS in breaststroke remain with the same distance for different stroke styles (e.g., 50 m) and could mean that despite their impairment, PS choose a race event based on the distance and stroke characteristics if the event is available for the impairment classification.

Regarding the IM, female and male PS had overlap with a variety of strokes (freestyle, breaststroke, and backstroke), all at the same 100 m distance. For NDS, the 200 m freestyle was the most commonly combined race event with the IM, which is consistent with previous findings that show NDS opting to swim this event in addition to their specialty (Dormehl and Williams, 2016).

These differences in race event involvement between PS and NDS could be due to several factors. Mainly, there are fewer swimmers in the PS race events and a fewer race events available to each of the PS classes leading to less concentrated competitions than there are in NDS competitions. The consequence of this low competition is that PS could be involved in different stoke style race events to reach a podium even if it is not their specialization. For the same number of performances, PS swimmers are half the number of NDS swimmers. Additionally, it could be due to the use of classifications, which aim to enable fairness between swimmers (Wu and Williams, 1999; Burkett et al., 2018; Payton et al., 2020) but also lead to a reduced number of swimmers in each race event. For example, in the international para competitions, the low numbers of swimmers in some race events means that there are not enough competitors to warrant heats and therefore swimmers often advance directly to finals (Fulton et al., 2009) and possibly reach the podium.

### The Performance Levels and the Impairment Distributions

Another important finding of this study was that the high-performance levels were associated with PS who were involved in multiple race events. Firstly, the difference between the mean performance level and the best performance level was greater and had a wider standard deviation for PS compared to NDS, demonstrating a less competitive field overall with a wider range of capabilities for PS. Between sexes, the difference between the mean and best performances was larger for female PS compared with male PS, reflecting a more competitive field among males. This difference between sexes was less pronounced for the NDS. These two finding may indicate a distinction: NDS are specialized in a swim stroke, distance, or race event based on their characteristics; The PS, despite their stroke characteristics, are willing to participate in secondary events because the low number of swimmers could allow to succeed in an event in which they are not specialized if the race is available to their impairment class.

Para swimmers follow traditional periodized patterns of variable training volume before important competitions similar to those observed in competitive swimmers competing at the Olympic level (Fulton et al., 2010) and the staff, coaches and athletes are more and more in a process of professionalization (Fulton et al., 2010). However, due in large part to the impact of impairment, performance levels between PS and NDS do not seem to be comparable. This is true across several sports (Grobler et al., 2015; Burkett et al., 2018; Schipman et al., 2019). In swimming, PS with minimal impairments still have performance detriments in comparison with their NDS counterparts (Taylor et al., 2016). This difference can be found right from the swim-start (Dingley et al., 2014).

Overall, PS involved in one race event showed no significant performance difference compared to swimmers involved in two race events. This shows that the best performances were achieved by swimmers who swam in several race events. Indeed, PS who performed in more than five race events had a significantly higher performance level than others. Specifically, for the freestyle race events, the difference between the mean performance and best performance was smaller and could be explained by a higher competition concentration of PS. For both male and female PS in breaststroke and male PS in backstroke, the difference between the mean and best performances was larger, showing a more heterogeneous level across the competitors. The Para swimmers who swam breaststroke were the least likely to be involved in more than five race events and who did not perform better for those who did more than five race events., highlighting the increased specificity for this stroke. This could be explained by the technical specificity of breaststroke (Mooney et al., 2015) and a greater number of swimmers from impairment classifications 7, 8, and 9.

The majority of NDS swam between 1 and 2 race events, which resulted in the highest level of performance. Except for men's freestyle and the category where we combined all strokes, swimmers who swam in two race events and more performed significantly better than those who have been involved in one race event. Specialization (Yustres et al., 2019; Staub et al., 2020) among NDS at an early age, which can optimize morphological characteristics (Pla et al., 2019), could explain the differences. However, this is still strongly debated. For the IM, similar trends were observed with higher performance levels existing based on the number of events. Moreover, none of the PS participated exclusively in this event, showing no specialization for this race event.

The impact of impairment on swimming performance is well-demonstrated (Wu and Williams, 1999; Burkett et al., 2018) and appears to be consistent with the impairment classification distribution of the top 100 swimmers per event in this study. By selecting the top 100 Para swimmers, the lower classes 1–6 were inevitably exclude from the analysis whereas the classes 9, 10, 13, and 14 were the most represented. Indeed, PS from the classifications 9–14 experience the lowest passive drag (Oh et al., 2013), a greater functional ability despite activity limitations for swimmers with limb deficiencies (Hogarth et al., 2018), fewer musculoskeletal limitations, and also benefit more substantially from traditional strength and conditioning sets, skill drills, and pacing strategies (Fulton et al., 2009). In the swim-start phases, swimmers with no physical disabilities were significantly faster compared to swimmers with physical disabilities (Dingley et al., 2014), which were well-represented in this study by swimmers in the classifications 13 and 14. Para swimmers may improve more through training optimization that is developed for their impairment level and targeted at developing muscle mass and upper-body power (Dingley et al., 2015).

### Age

The results showed that female PS were generally younger than female NDS, while there were few significant differences observed among males. The mean age of the top 100 NDS were consistent with previous studies on age-related swimming performance and relatively young compared to other sports which is confirmed by the scientific literature (Allen and Hopkins, 2015; Knechtle et al., 2016; Marck et al., 2019). However, this study didn't analyze the age-peak performance.

However, for PS, these findings should be considered according to the impairment type, the origin and development of the disability, either born with it or acquired later in life. Indeed, most of the swimmers in the study have slight disabilities. It would be interesting to have this information in order to study the time required to reach the maximal potential capacity and understand the mean age of the PS.

### Study Limitations

Not all race events were swum by all swimmers, which did not allow us to analyze the performance level by specific race event and instead we were limited to the cumulative events across a stroke. Due to a lack of data and the sampling method, only the PS with no or light impairment were represented in the top 100 and as a consequence, swimmers with a high degree of impairment (1–6) were not represented whether over half of the sports classes. Furthermore, the longest distance swam by the PS was the 400 m, so it is difficult to conclude if specialization occurs by distance.

### Perspectives

It would be interesting to further these analyses to all swimmers who participate in the Paralympic Games. However, the amount of data available is currently insufficient.

The results may also highlight the fact that it could be necessary to participate in several race events to increase the chances of victory depending on the level of competition in any specific race event. Indeed, it may be useful to analyze the level of competition in certain events to try to compete even if it is not the swimmer's specialty.

## Conclusion

Para swimmers showed a higher involvement in multiple race events compared to NDS. The PS competed across all race events while the NDS appeared to be more specialized in 1 or 2 strokes over several distances. The more race events in which PS took part, the higher the mean level of performance compared with the top performer. The greatest difference between the mean performance and the best performance among PS was seen when there was a less competitive field, which was linked to a lower number of international PS. The lower number of competitors could be explained by fewer people practicing and competing due to a variety of difficulties that come with swimming. In this context, PS are capable of competing in many race events, while impairment classifications allow the race event to remain competitive with regard to swimmers' disabilities.

## Data Availability Statement

Publicly available datasets were analyzed in this study. This data can be found at: https://drive.google.com/file/d/1Lzem2o74dWRQKTHHDDP9uw_3wbeNw996/view?usp=sharing.

## Ethics Statement

This study was designed and monitored by the IRMES (Institut de Recherche bio-Médicale et d'Epidémiologie du Sport) scientific committee. It used a research protocol classified as non-interventional, in which all acts are performed in a normal manner, without any supplemental or unusual procedure of diagnosis or monitoring. (Article L1121–1 of the French Public Health Code).

## Author Contributions

JS, GS, BL, AM, NF, J-FT, and AS conceived and designed the research. JS, GS, BL, AM, J-FT, and AS performed and analyzed the research. JS and GS wrote the manuscript. All authors read and approved the final manuscript.

## Conflict of Interest

The authors declare that the research was conducted in the absence of any commercial or financial relationships that could be construed as a potential conflict of interest.
